# Nanocellulose/two dimensional nanomaterials composites for advanced supercapacitor electrodes

**DOI:** 10.3389/fbioe.2022.1024453

**Published:** 2022-10-04

**Authors:** Qidi Liang, Yaxuan Wang, Yanfan Yang, Ting Xu, Ying Xu, Qingshuang Zhao, Su-Hak Heo, Min-Seok Kim, Young-Hwan Jeong, Shuangquan Yao, Xueping Song, Sun-Eun Choi, Chuanling Si

**Affiliations:** ^1^ Tianjin Key Laboratory of Pulp and Paper, Tianjin University of Science and Technology, Tianjin, China; ^2^ Guangxi Key Laboratory of Clean Pulp & Papermaking and Pollution Control, College of Light Industry and Food Engineering, Guangxi University, Nanning, China; ^3^ Department of Medicinal Bioscience, Konkuk University (Glocal Campus), Chungju-si, Chungcheongbuk-do, South Korea; ^4^ Department of Forest Biomaterials Engineering, College of Forest & Environmental Sciences, Kangwon National University, Chuncheon, South Korea; ^5^ State Key Laboratory of Tree Genetics and Breeding, Northeast Forestry University, Harbin, China

**Keywords:** supercapacitor, nanocellulose, MXene, graphene, electrode

## Abstract

With the emerging of the problems of environmental pollution and energy crisis, the development of high-efficiency energy storage technology and green renewable energy is imminent. Supercapacitors have drawn great attention in wearable electronics because of their good performance and portability. Electrodes are the key to fabricate high-performance supercapacitors with good electrochemical properties and flexibility. As a biomass based derived material, nanocellulose has potential application prospects in supercapacitor electrode materials due to its biodegradability, high mechanical strength, strong chemical reactivity, and good mechanical flexibility. In this review, the research progress of nanocellulose/two dimensional nanomaterials composites is summarized for supercapacitors in recent years. First, nanocellulose/MXene composites for supercapacitors are reviewed. Then, nanocellulose/graphene composites for supercapacitors are comprehensively elaborated. Finally, we also introduce the current challenges and development potential of nanocellulose/two dimensional nanomaterials composites in supercapacitors.

## 1 Introduction

**FIGURE 1:**
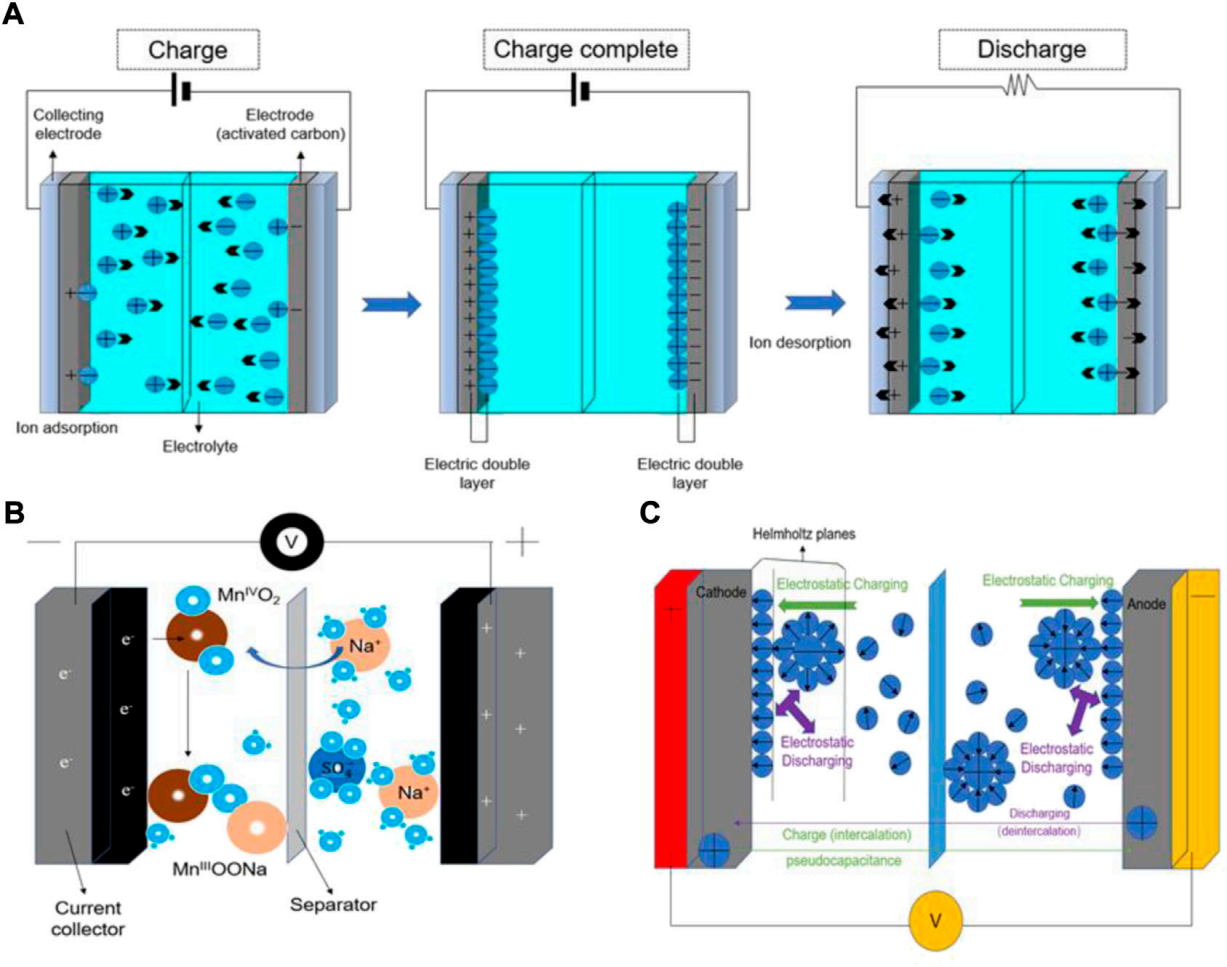
The working mechanism of supercapacitors. **(A)** Working principle of EDLC during charging and discharging, **(B)** pseudocapacitor, and **(C)** hybrid capacitor.

The emission of greenhouse gases and the depletion of fossil fuels have led to an increasing global demand for renewable energy, which has promoted human beings to invest more and more funds in the development, utilization, and storage of new energy (Gross et al., 2003; Lund, 2007; Si et al., 2009a; Si et al., 2013a; Du et al., 2016; Dai et al., 2019; Yang et al., 2019a; Yang et al., 2020; Liu et al., 2021a; Xiong et al., 2021a; Huang et al., 2021; Liu et al., 2022a; Xu et al., 2022). As an advanced energy storage technology, supercapacitors have been widely used in portable electronic products, memory backup, electric vehicles, and military equipment, due to their environmental friendliness, high power density, maintenance-free, and long-life properties (Si et al., 2008; Si et al., 2009b; Si et al., 2013b; Dubal et al., 2015; Salunkhe et al., 2016a; Salunkhe et al., 2016b; Salunkhe et al., 2017; Xu et al., 2021a; Liu et al., 2021b; Xiong et al., 2021b; Liu et al., 2021c; Liu et al., 2021d; Ma et al., 2021). Hybrid capacitors, pseudocapacitors, and Electric double-layer capacitors (EDLCs) can be distinguished depending on the storage mechanism or cell configuration. The key characteristic of EDLCs is the double-layer capacitance, for example, it generates at the interface between the liquid electrolyte and the adjacent conductive electrode. At this boundary, two layers of charges of opposite polarity are formed, one in the electrolyte and the other on the electrode surface (Zhang and Zhao, 2009; Zhai et al., 2011; Qiang et al., 2021; Zhang et al., 2021). Figure 1A shows the working mechanism of EDLCs during the charging and discharging process. When charging, the positive and negative electrodes generate a stable electric field. The cathode attracts the anions in the electrolyte, and the anode attracts the cations in the electrolyte to generate an electric double layer; when discharging, the anions and cations in the electrolyte return to the electrolyte to form an electrically neutral solution. As shown in Figure 1B, pseudocapacitors store charges through redox reactions, electrosorption, or intercalation mechanisms (Conway et al., 1997; Zhao et al., 2011; Augustyn et al., 2014). These electrochemical reactions allow pseudocapacitors to achieve higher specific capacitance than EDLCs. Hybrid capacitors have two different electrodes, one electrode mainly exhibits electrostatic capacitance and the other electrode mostly exhibits electrochemical capacitance (Figure 1C) (Ma et al., 2007).

**FIGURE 2:**
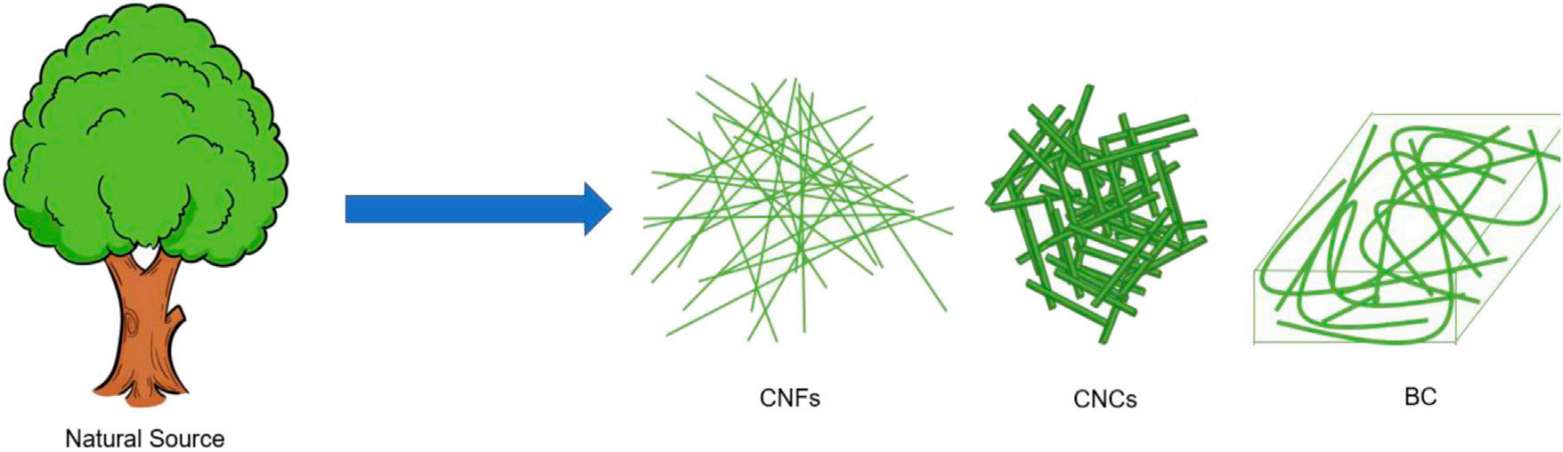
Schematic diagram of the classification of nanocellulose.

Cellulose, as the most abundant natural polymer on Earth, consists of hundreds of strands β (1 → 4) linked D-glucose units (Updegraff, 1969; Crawford, 1981; Hu, et al., 2019; Liu et al., 2021e; Liu et al., 2022b). Nanocellulose has been extensively studied as the rapid development of nanotechnology. Nanocellulose is a kind of polymer that reduces the size of cellulose in a certain dimension to the nanometer level through chemical, physical and biological methods (Figure 2) (O’sullivan, 1997; Lu et al., 2019; Du et al., 2019; Du et al., 2021a Du et al., 2021b; Du et al., 2022; Liu et al., 2020a; Xu et al., 2020a; Xu et al., 2021b; Xu et al., 2020b; Xu et al., 2020c). In addition to the characteristics of cellulose, nanocellulose also demonstrates large aspect ratio, and specific surface area, excellent mechanical strength, and good chemical reactivity (Dai et al., 2017; An et al., 2019; Li et al., 2019; Li et al., 2020a; An et al., 2020; Wang et al., 2020a; Dai et al., 2020; Si and Xu, 2020; Liu et al., 2021e; Wang et al., 2021; Wang et al., 2022). According to the morphological structure, nanocellulose can be divided into three classes: bacterial cellulose (BC), cellulose nanofibers (CNFs), and cellulose nanocrystals (CNCs) (Ostadhossein et al., 2015; Tayeb et al., 2018; Xie et al., 2018; Xie et al., 2019; Chen et al., 2020a; Chen et al., 2020b; Lu et al., 2020; Li et al., 2022a). Nanocellulose could be mixed with other conductive materials as electrode materials for supercapacitors (Kang et al., 2012; Li et al., 2018; Nie et al., 2018; Zhang et al., 2018; Li et al., 2020b; Liu et al., 2020b; Li et al., 2022b).

**FIGURE 3:**
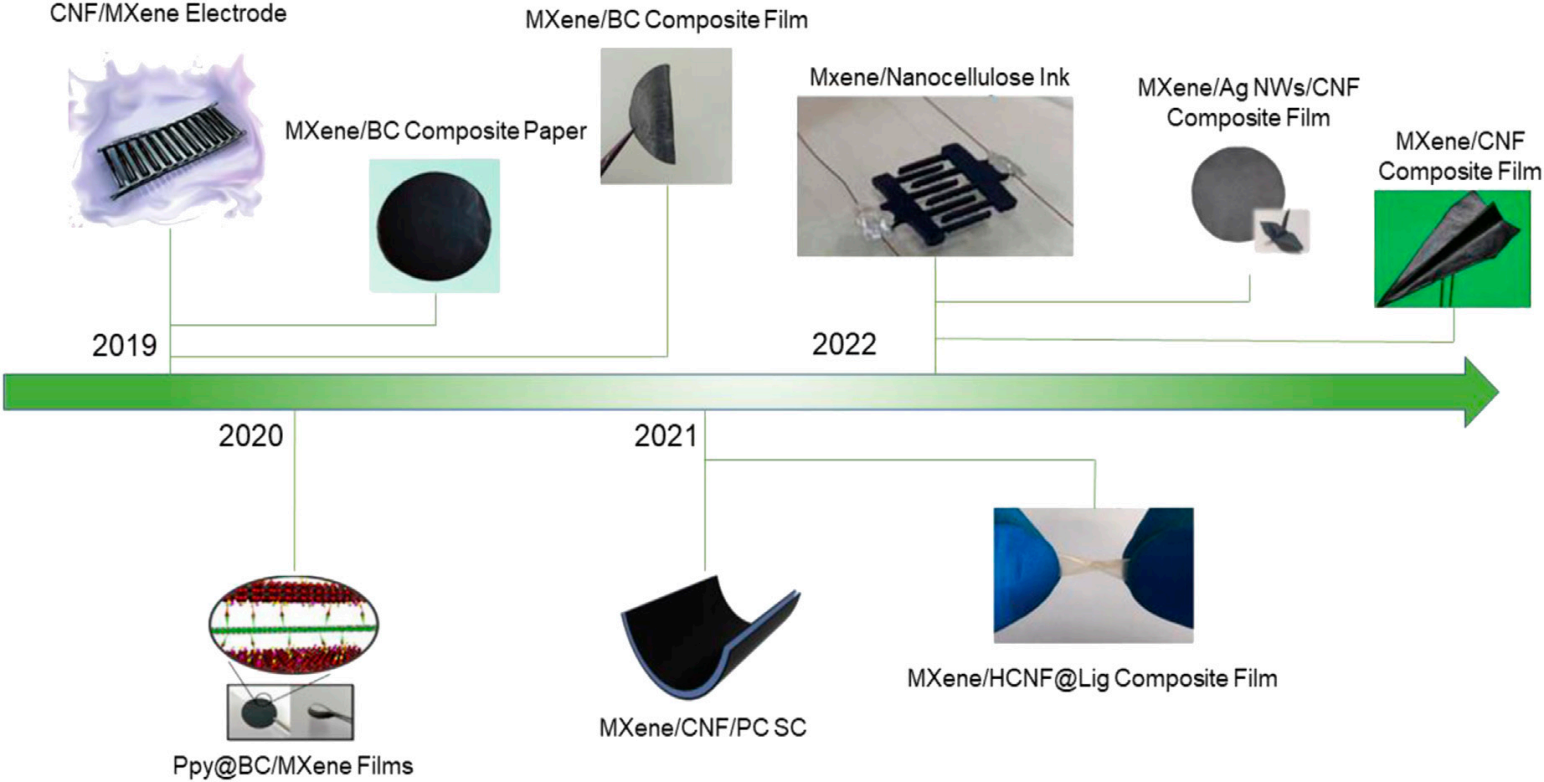
Brief timeline of nanocellulose/MXene composites for supercapacitors. Reproduced from: Tian et al. (2019), Wiley-VCH; Jiao et al. (2019), Wiley-VCH; Wang et al. (2019), Wiley-VCH; Song et al. (2020), Springer Nature; Chen et al. (2021a), Elsevier; Chang et al.(2021), Royal Society of Chemistry. Zhou et al. (2022), Wiley-VCH; Tang et al. (2022), Wiley-VCH; Chen et al. (2022), Elsevier.

**FIGURE 4:**
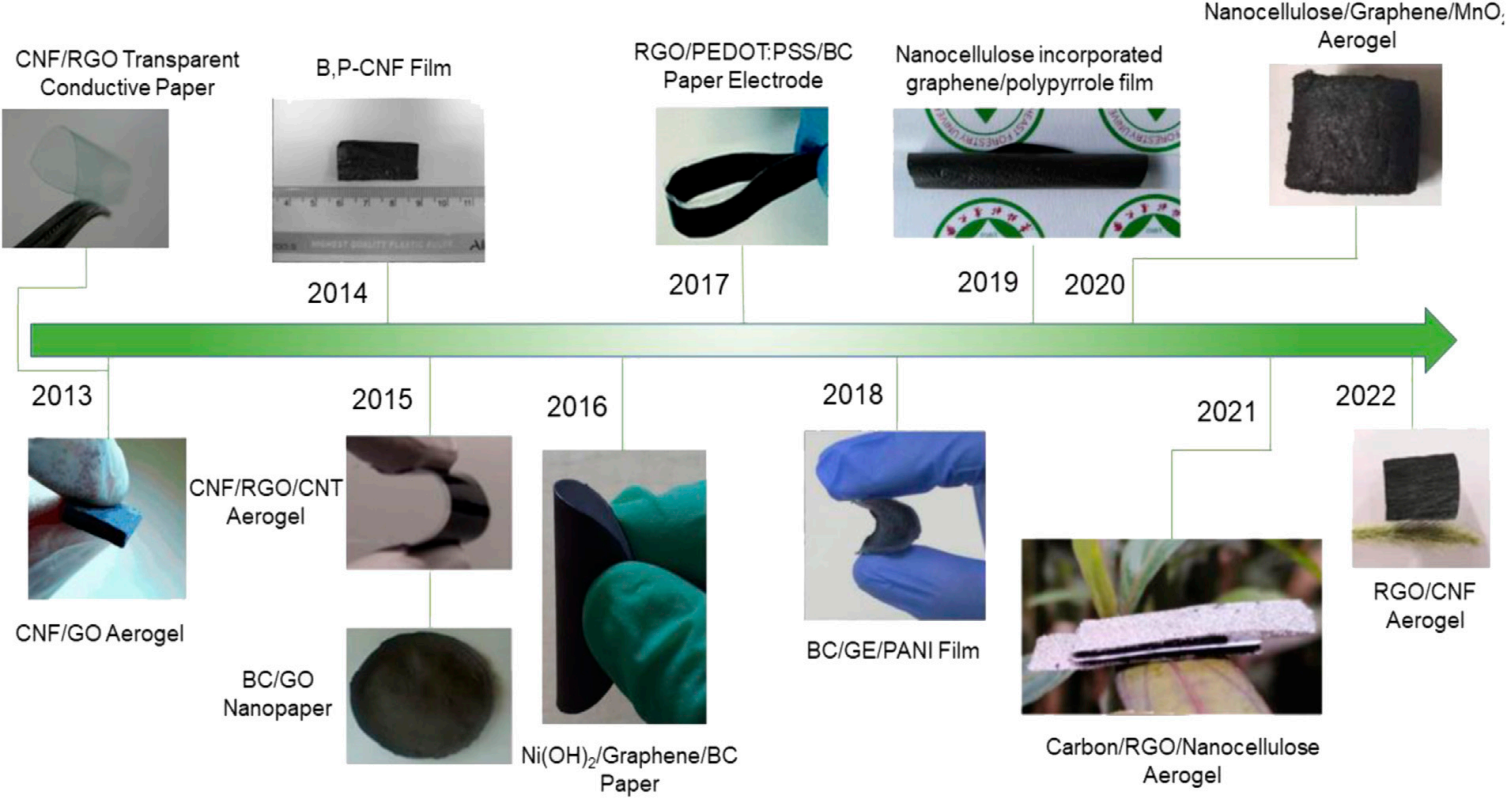
Brief timeline of nanocellulose/graphene composites for supercapacitors. Reproduced from: Gao et al. (2013b), Elsevier. Hamedi et al. (2013), Wiley-VCH; Chen et al. (2014), Wiley-VCH; Zheng et al. (2015), American Chemical Society; Liu et al. (2015), Royal Society of Chemistry. Ma et al. (2016a), Elsevier. Zhao et al. (2017), American Chemical Society; Luo et al. (2018), Elsevier. Hou et al. (2019), Elsevier. Wang et al. (2020b), Springer; Chen et al. (2021b), Elsevier; Liu et al. (2022c), Springer.

Conductive materials are essential in the preparation of energy storage equipments and are considered objects that permit the flow of electrical current. Both MXene and graphene are emerging two-dimensional materials that have been successfully used for supercapacitors as electrode materials. However, the high cost of preparing graphene and easy aggregation affects the surface wettability and dispersibility of graphene in the electrolyte, which reduces the electrical conductivity and thus reduces the effective specific surface area. In the same way, the large van der Waals forces between layers of MXene cause the expansion of inactive pores, limiting the transfer of electrolyte ions, resulting in the decreasing of the electrical conductivity. The introduction of nanocellulose can avoid graphene aggregation and enlarge the active pores between the MXene sheets. At the same time, due to its good properties, including surface wettability, flexibility, high mechanical strength, thermal stability, and hydrophilicity, biomass raw materials can be converted into value-added products, which could promote the economic cycle. Therefore, the composite of nanocellulose with graphene and MXene is the promising electrode materials for supercapacitors (Gao et al., 2013a; Li et al., 2014; Yan et al., 2014). Figures 3, 4 show the recent development of composites (MXene/CNF and graphene/CNF) as electrodes for supercapacitors, respectively.

## 2 Nanocellulose/two dimensional nanomaterials composites

### 2.1 Nanocellulose/MXene composites for supercapacitors

#### 2.1.1 MXene and composite strategy

MXene, comprising of transition metal nitrides, carbides, or carbonitrides, is a class of two-dimensional inorganic compounds. It was first reported in 2011 that MXene materials possess metallic conductivity of transition metal carbides due to hydroxyl groups or terminal oxygens on its surface (Naguib et al., 2011; Naguib et al., 2014). Generally, MXene is prepared by selectively etching the A layer from the MAX phases, and the etching solution contains fluoride ions, such as hydrofluoric acid (HF) (Naguib et al., 2014), ammonium bifluoride (NH_4_HF_2_) (Halim et al., 2014) or hydrochloric acid (HCl), and lithium fluoride (LiF) (Ghidiu et al., 2014). Because of the strong interlayer van der Waals interaction, MXene is easy to stack, which hinders the effective utilization of surfactant sites and the rapid transport of ions (Yang et al., 2019b). To solve the above problems, the “spacer” is needed to increase the space between the MXene sheets, thereby increasing the surfactant sites to achieve effective ion migration.

**FIGURE 5:**
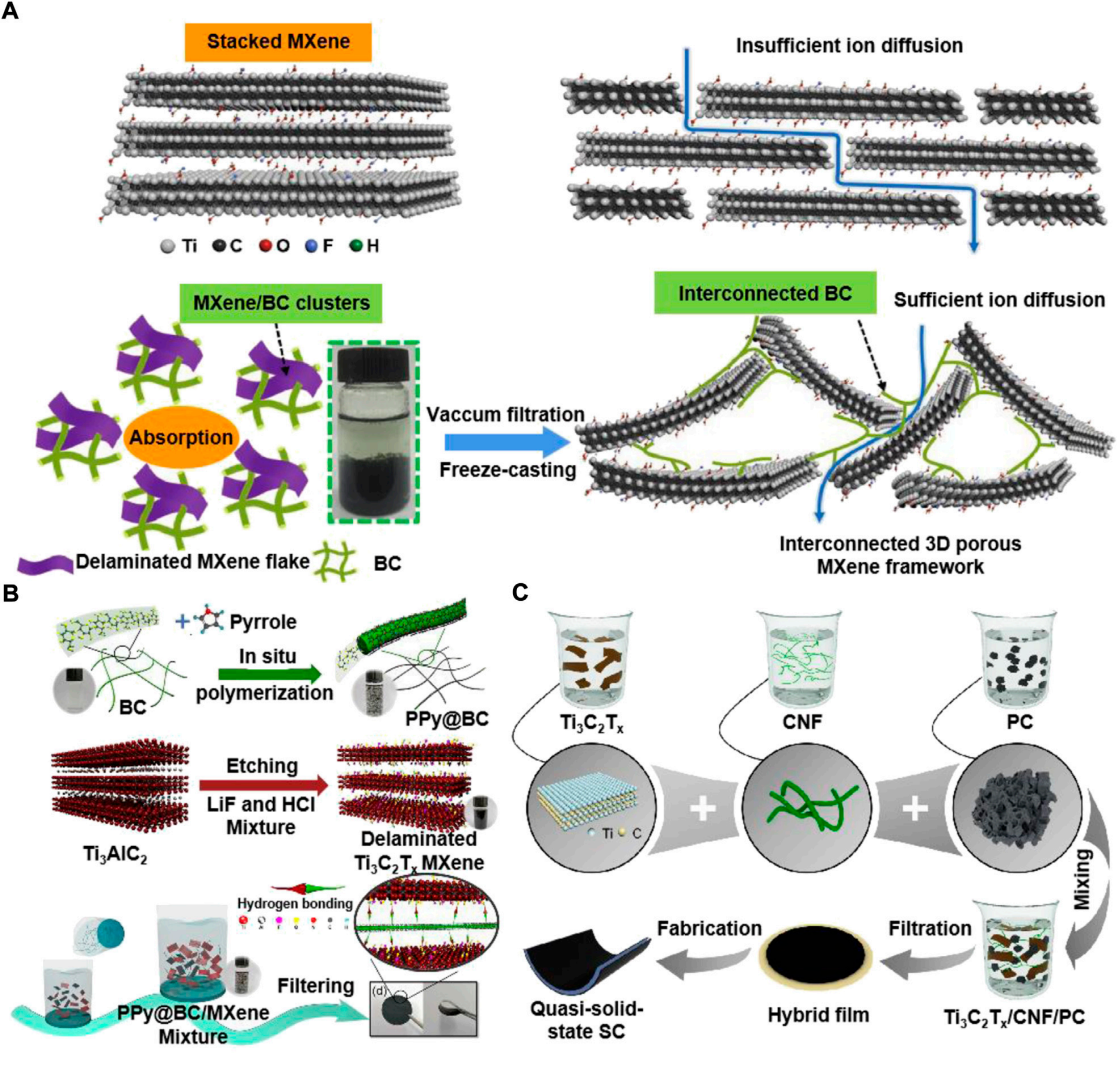
The preparation process of the **(A)** porous MXene architecture, **(B)** PPy@BC/MXene composite paper, and **(C)** Ti_3_C_2_T_x_/CNF/PC hybrid film. Reproduced from: **(A)** Wang et al. (2019), Wiley. **(B)** Song et al. (2020), Springer. **(C)** Chen et al. (2021a), Elsevier.

Nanocellulose is one-dimensional fibrous structure, that can act as a “spacer” and improves ion transport across the electrode/electrolyte interface. For example, Tian et al. (2019) prepared a two-dimensional film composite of nanocellulose and MXene for supercapacitor electrodes with high electronic conductivity (2.95 × 10^4^ S/m). This is because the small width of the CNFs (around 3.5 nm) makes it possible to have better conductivity between two-dimensional sheets. Simultaneously, since the addition of CNFs did not limit the ability of ion transport and pseudocapacitance storage, its gravimetric capacitance was as high as 298 F/g. As shown in Figure 5A, Wang et al. (2019) reported 3D porous BC/MXene self-supporting film using a simple and time-saving method. The prepared BC/MXene film has a good hierarchical porous structure, excellent mechanical properties, and high flexibility and showed ultra-high capacitance of 416 F/g. In addition, the assembled asymmetric supercapacitor with negative electrode of BC/MXene film and positive electrode of BC/polyaniline (PANI) had a high energy density (252 μWh/cm). This work developed a simple method to assemble high performance 3D porous films electrodes of advanced energy storage devices with 2D MXene materials.

#### 2.1.2 Nanocellulose/MXene-based supercapacitors

So far, investigation on nanocellulose-MXene based electrodes for supercapacitors with basic compositions and electrochemical performance have been carried out, as showed in Table 1 in literatures. It can be seen that the future study of nanocellulose and MXene composite materials as electrodes for supercapacitors can be divided into two directions.

**TABLE 1:** Electrochemical performance of the nanocellulose/MXene-based supercapacitors.

Materials	Electrolyte	Current density	Specific capacitance	Energy density	Power density	Cycle life-capacitance retention	References
CNFs/MXene	H_2_SO_4_	0.57 mA/cm^2^	25.3 mF/cm^2^	0.08 μ Wh/cm^2^	145.00 μ Wh/cm^2^	10000-86.8%	Tian et al. (2019)
BC/MXene	H_2_SO_4_	3.00 mA/cm^2^	87.0 F/g	252.00 μ Wh/cm^2^	2.12 mV/cm^2^	10000-96.5%	Wang et al. (2019)
PPy@BC/MXene	H_2_SO_4_	1.00 mA/cm^2^	294.0 F/g	33.10 Wh/kg	243.00 w/kg	10000-83.5%	Song et al. (2020)
CNFs/PC/MXene film	KOH	0.10 mA/cm^2^	143.0 mF/cm^2^	2.40 μ Wh/cm^2^	17.50 μ W/cm^2^	10000-90.0%	Chen et al. (2021a)
CNFs@sodium lignosulfonate/MXene	PVA-H_2_SO_4_	0.5 A/g	248.0 F/g	16.20 Wh/L	633.10 W/L	10000-86.5%	Chang et al. (2021)
CNFs/MXene@SnS_2_	PVA-H_2_SO_4_	1.00 mA/cm^2^	205.0 mF/cm^2^	6.70 μ Wh/cm^2^	1206.00 μ W/cm^2^	5000-87.4%	Cai et al. (2021)

One is to pursue higher performance supercapacitors, and the other is to develop multifunctional supercapacitors. Because of the potential synergistic effect, the composite hybrid materials of different compositions are expected to be an effective strategy. For example, Song et al. (2020) prepared an enhanced electrochemical performance freestanding electrode of polypyrrole (PPy)@BC/MXene composite film (Figure 5B). The BC as a template used for depositing PPy nanoparticles uniformly, and PPy@BC nanofibers can also be embedded in the MXene layer to effectively prevent the re-accumulation of MXene and expand its interlayer space, which provided a wide range of accessible electrochemical active sites. The prepared PPy@BC/MXene electrode showed good specific capacitance of 550 F/g and long cycle life (83.5% capacitance retention after 10,000 cycles). The assembled symmetric supercapacitor by PPy@BC/MXene electrodes exhibited a high energy density of 33.1 Wh/kg. As shown in Figure 5C, Chen et al. (2021a) prepared a porous flexible MXene/CNFs/porous carbon (PC) hybrid film through a simple method of vacuum filtration. Abundant micropores were provided by three-dimensional PC for charge storage and amount of meso/macropores were provided by three-dimensional PC for the rapid diffusion of ions. One dimensional CNFs enables the hybrid film to have high mechanical properties by combining adjacent MXene sheets and PC. Supercapacitors assembled with MXene/CNFs/PC films as electrodes showed high capacitance of 143 mF/cm^2^ and high energy density of 2.4 μWh/cm^2^.

**FIGURE 6:**
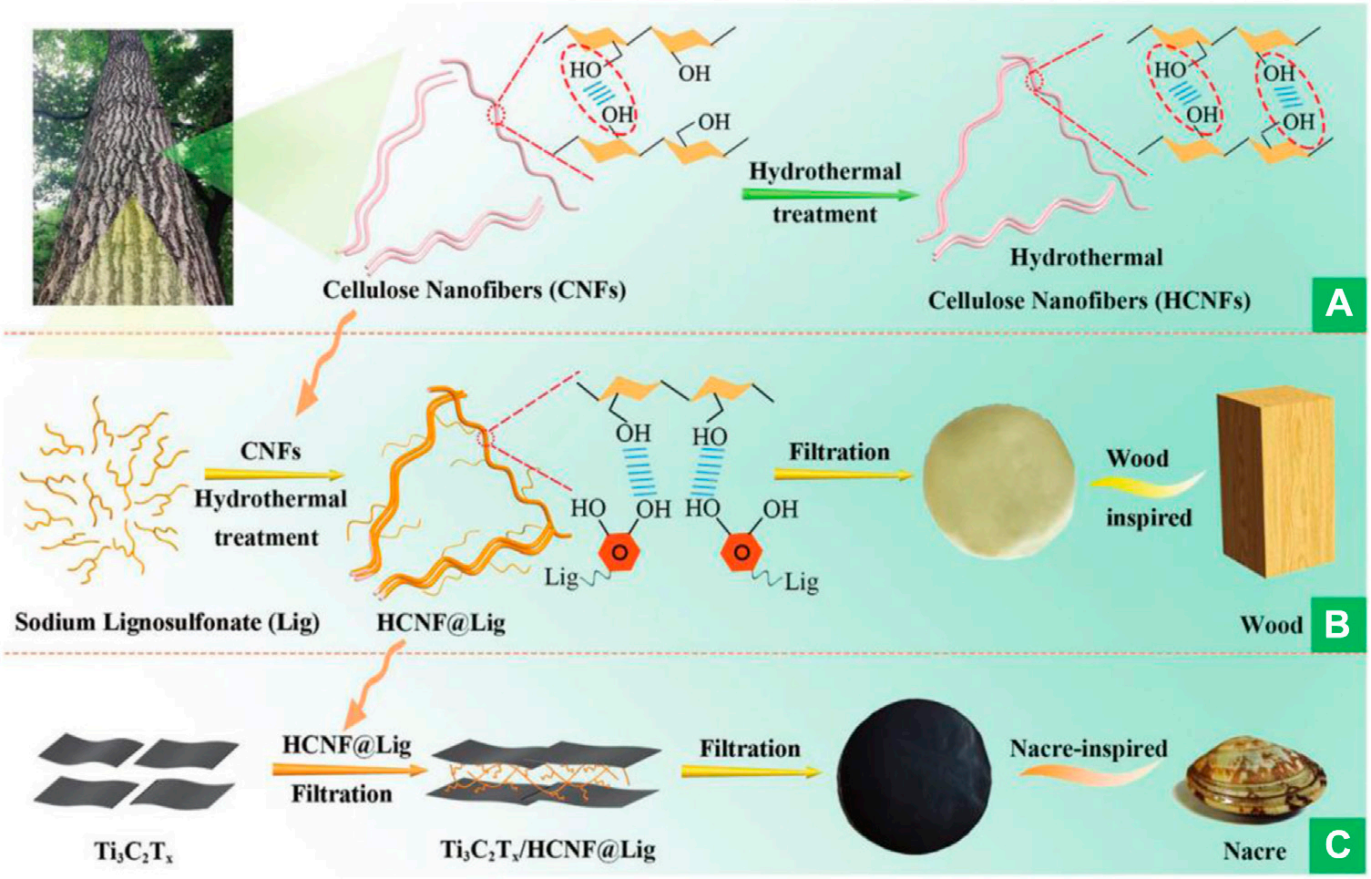
The preparation process of the nacre-inspired MXene/HCNF@Lig composite films. Reproduced from: Chang et al. (2021), Royal Society of Chemistry.

More rational dimensional structure design should be explored. For example, to combine with MXene nanoflakes, Cheng et al. (2021) constructed one-dimensional conductive BC@PPy nanofibers with core-shell structure. The obtained MXene/BC@PPy film electrodes displayed high capacitance of 388 mF/cm^2^. Based on a two-dimensional film composite with nanocellulose and MXene, Cai et al. (2021) added SnS_2_ to assemble into a nacre-like structure material. The prepared composite material exhibited excellent mechanical strength (78.3 MPa) without sacrificing toughness. The supercapacitor electrodes based on this material maintained 91.5% capacitance, provided 6.7 μWh/cm^2^ energy density after 4000 cycles, and exhibited excellent flexibility (over 90% capacitance retention after 500 folding/unfolding cycles). Similarly, Chang et al. (2021) synthesized a nacre-like composite film obtained from MXene, CNFs, and lignosulfonate by a hydrothermal process (Figures 6A–C). The composite film exhibited good mechanical strength (114 MPa) compared with the CNFs film (95 MPa). The supercapacitor assembled by the composite films exhibited an excellent specific capacitance (748.96 F/cm^3^).

### 2.2 Nanocellulose/graphene composites for supercapacitors

#### 2.2.1 Graphene and composite strategy

**FIGURE 7:**
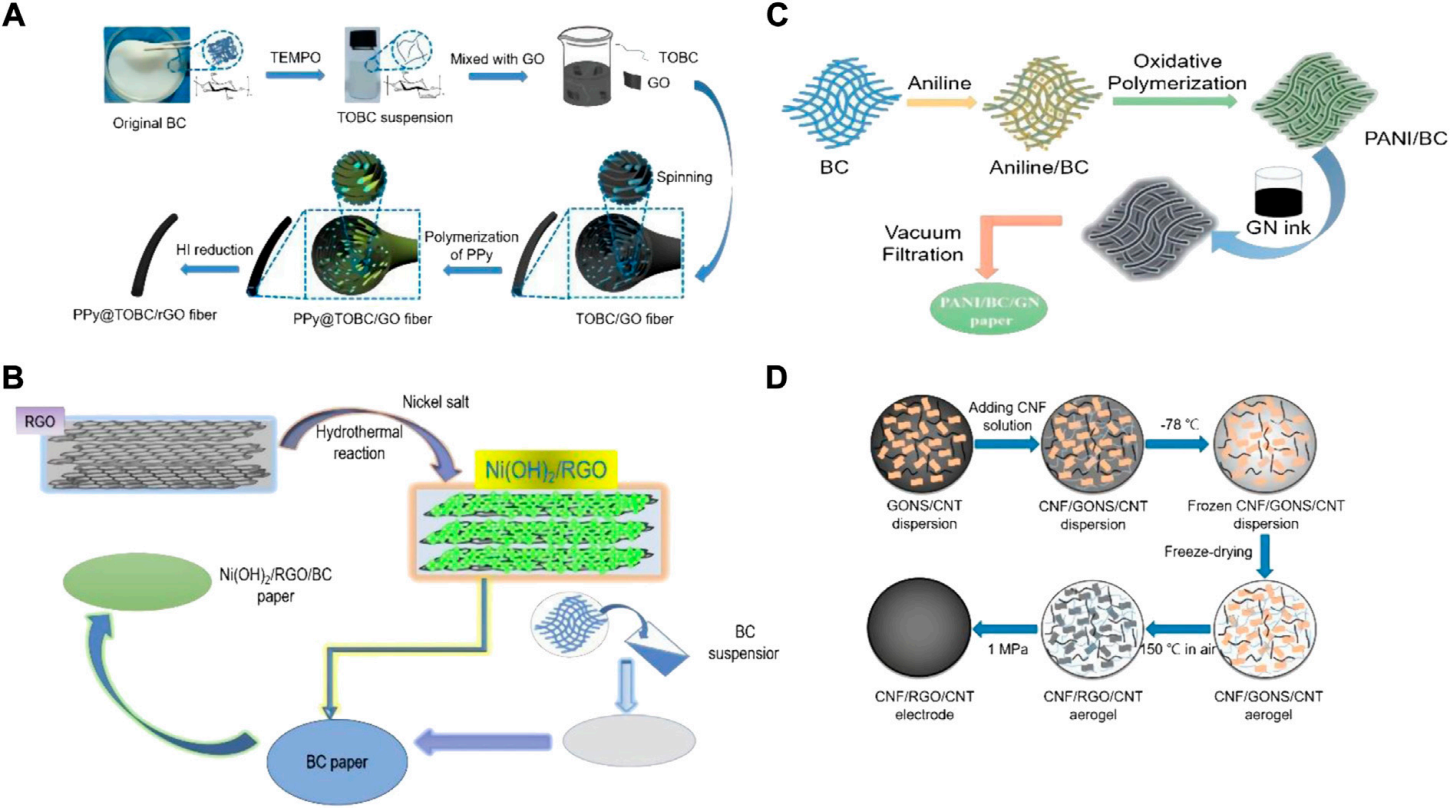
The preparation process of the **(A)** PPy@TOBC/rGO macrofibers, **(B)** Ni(OH)_2_/RGO/BC electrode, **(C)** PANI/BC/GN freestanding paper electrodes, and **(D)** CNF/RGO/CNT electrode. Reproduced from: **(A)** Sheng et al. (2019), Elsevier. **(B)** Ma et al. (2016a), Elsevier. **(C)** Liu et al. (2016), Royal Society of Chemistry. **(D)** Zheng et al. (2015), American Chemical Society.

Graphene has a hexagonal honeycomb lattice structure and is a two-dimensional nanomaterial composed of carbon atoms with sp2 hybrid orbitals. It has high electron mobility, high specific surface area, excellent mechanical properties, and stable chemical properties. It shows great potential for applications in photothermal conversion, electrochemical energy storage, electronic screens, and industrial catalysis (Novoselov et al., 2012; Ferrari et al., 2015). However, due to the easy accumulation of graphene, the dispersibility and surface wettability of graphene in the electrolyte are affected, and the electrical conductivity is greatly affected. In the composite process of nanocellulose and graphene, nanocellulose acts as a nano-spacer layer, providing a continuous conductive path between different graphene nano-sheet layers and effectively preventing graphene aggregation. This change solves the problem of ion diffusion difficulty in graphene-based materials in electrolytes (Gao et al., 2013a). Table 2 shows the performance of composite materials based on nanocellulose and graphene as electrodes for supercapacitors in recent years. Recent research on supercapacitors mainly focuses on improving electrochemical performance. Nanocellulose-graphene based materials are suitable for selecting electrode materials for high-energy and high-power density supercapacitors. Sheng et al. (2019) constructed a flexible, high-performance fiber-based supercapacitor with synthesized PPy, 2,2,6,6-Tetramethylpiperidine-1-oxyl (TEMPO)-oxidized BC (TOBC), and graphene oxide (GO) into a fiber by wet spinning (Figure 7A). The assembled supercapacitor showed excellent electrochemical performance because TOBC can prevent the aggregation of graphene nanosheets. Carboxylic acid groups and pyrrole monomers penetrated the fiber’s interior, resulting in the generation of PPy inside the fiber, and this unique layered structure provided excellent electrochemical stability. The fiber-based supercapacitor exhibited a high energy density (8.8 mWh/cm^3^).

**TABLE 2:** Electrochemical performance of the nanocellulose/graphene-based supercapacitors.

Electrodes (electrolyte)	Voltage (V)	Specific capacitance (F g^−1^)	Energy density (mWh g^−1^)	Power density (mW g^−1^)	Cycle life-capacitance retention	References
BC/GO (1 M H_2_SO_4_)	1.2	160.0	—	—	2000-90.3%	Liu et al. (2015)
PPy/BC/RGO (1 M H_2_SO_4_)	1.0	271.0	5.75	587.5	8000-73.5%	Ma et al. (2016b)
PANI/BC/Graphene (1 M H_2_SO_4_)	0.8	477.0	4.25	930.0	8000-56.3%	Liu et al. (2016)
Ni(OH)_2_/RGO/BC (2 M KOH)	0.5	877.1	—	—	15000-93.6%	Ma et al. (2016a)
CNFs/CNTs/RGO (PVA/H_2_SO_4_)	1.0	252	7.10	2375.0	1000-99.5%	Zheng et al. (2015)
HRGO/BC (PVA/H_3_PO_4_)	0.4	65.9	9.20	112.9	5000-88%	Guan et al. (2019)
TOCN/RGO (1 M H_2_SO_4_)	0.5	398.5	—	—	10000-99.77%	Yang et al. (2019c)
PPy/Graphene/Cellulose (1 M H_2_SO_4_)	1.0	630	—	—	—	Aphale et al. (2015)
PPy/TOBC/RGO	0.5	391	4.10	429.3	5000-79%	Sheng et al. (2019)

#### 2.2.2 Nanocellulose/graphene-based supercapacitors

Nanocellulose-based two-dimensional paper or film have attracted more and more attention in the field of flexible electrodes for supercapacitors. Vacuum filtration is one of the most common methods to prepare nanopaper or composite film. For instance, Ma et al. (2016a) coated graphene-wrapped Ni(OH)_2_ on BC to prepare a flexible film using the hydrothermal method and filtration technology (Figure 7B). This method provided an effective pore volume for high-quality loading and provided a fast channel for ion and electron transport. The prepared film electrode achieved a significant area capacitance of 10.44 F/cm^2^ under a load of 11.9 mg/cm^2^ and had a capacitance retention rate of 93.6% after 15000 cycles. Later, they used simple *in-situ* polymerization and filtration methods to prepare a freestanding conductive film from PPy/BC composite nanofibers combined with graphene (Ma et al., 2016b). The introduction of graphene effectively solves the problem of poor conductivity of PPy/BC to ensure high electron and ion transfer. The prepared flexible electrode showed good flexibility and could be bent to a large extent.

The symmetric supercapacitor made of two PPy/BC/RGO paper electrodes exhibited a large area capacitance (1.67 F/cm^2^) and a high energy density of 0.23 mWh/cm at a power density of 23.5 mW/cm^2^. The excellent electrochemical performance made it a promising candidate for flexible energy storage devices. Similarly, Liu et al. (2016) prepared a flexible film electrode using PANI, BC and graphene by a simple *in-situ* chemical polymerization and vacuum filtration method, as shown in Figure 7C. The prepared film electrode possessed a regular interconnected pore channel network, which not only contributed to electron transport and ion dispersion in the whole interconnected network, but also overcame the aggregation of graphene and PANI/BC in three-dimensional conductive paper. The symmetric supercapacitor assembled by the film electrodes can provide high area capacitance (1.93 F/cm^2^) and energy density (0.17 mWh/cm^2^).

Liu et al. (2015) constructed BC/GO nanopaper by covalently intercalating GO and BC fiber through one-step esterification. The tensile strength of the prepared reinforced BC/GO composite increased by 12.2 times and the elongation at break increased by 20.9% compared with the original GO. The prepared composite showed excellent conductivity (171 S/m) and high specific capacitance (160 F/g) and had an outstanding capacitance retention of 90.3% after 2,000 cycles. Compared with the original graphene, porous graphene has a multi-layered pore structure on the graphene base surface, which can not only promote the storage and diffusion of electrolyte ions, but also enhance the electrochemical performance of graphene-based electrode materials. Guan et al. (2019) prepared holey GO (HRGO)/BC composite film materials with porous structure, good wettability, and excellent mechanical flexibility through biological assembly method. HRGO/BC composite film has good foldability and can produce high tensile strength of 204 MPa and elongation of 13.8%. The symmetric supercapacitor assembled by HRGO/BC composite film electrode showed a specific capacity (65.9 F/g) and an energy density (9.2 Wh/kg).

Electrode materials with three-dimensional network structure and high conductivity are the keys to developing high-performance supercapacitors. Therefore, three-dimensional aerogel with porous structure has gradually become a popular candidate for supercapacitor electrode materials. Zheng et al. (2015) used CNFs, RGO, and carbon nanotubes (CNTs) to synthesize aerogels as electrodes (Figure 7D). The assembled supercapacitors exhibited suitable areal capacitance, areal power density, and energy density of 216 mF/cm^2^, 9.5 mW/cm^2^, and 28.4 μWh/cm^2^, respectively. Yang et al. (2019c) used TEMPO-oxidized CNFs (TOCN) and GO as precursors to prepare carbon aerogel (TOCN/RGO) by ion exchange, freeze drying and high-temperature carbonization. The obtained carbon aerogel electrode showed a high specific capacitance (398.5 F/g). At the same time, the carbon aerogel electrode also had excellent capacitance retention of 99.77% after 10,000 charge and discharge cycles. In addition, Gao et al. (2013a) used CNFs/RGO hybrid hydrogel as raw material to prepare hybrid aerogel by supercritical CO_2_ drying, and used it as electrode material for all-solid-state flexible supercapacitor. CNFs were used as nano spacers and electrolyte nano reservoirs of hybrid aerogel. The results showed that the supercapacitor had excellent surface capacitance (207 F/g) and energy density (20 mWh/cm^2^).

## 3 Conclusion

Most research on nanocellulose-based supercapacitors mainly concentrated on nanocellulose as a spacer or substrate in composites. As mentioned above, the electrodes of supercapacitors can be a composite of nanocellulose and other conductive materials. Currently, it is still a challenge to produce supercapacitors with high energy density and high capacitance. Therefore, to further improve the electrochemical performance of electrode materials, researchers need to add other materials (such as polypyrrole, sodium lignin thiosulfonate, etc.) to nanocellulose/MXene and nanocellulose/graphene composites. In addition, the composite structure can be changed to obtain different properties. For graphene/MXene and nanocellulose composites, improving the synergy between composites by changing the structure, morphology, distribution, and number of components is the focus of future research (Klemm et al., 2005; Panwar et al., 2011; Liu et al., 2021f; Liu et al., 2022d; Liu et al., 2022e; Liu et al., 2022f; Liu et al., 2022g).
